# Supplementary material for: Epidemiology of dengue, chikungunya and Zika virus infections in travellers: A 16-year retrospective descriptive study at a tertiary care centre in Prague, Czech Republic
Source: PLoS One. 2023 Feb 21;18(2):e0281612. doi: 10.1371/journal.pone.0281612 (PMC9942961; doi:10.1371/journal.pone.0281612)
Supplement: Attachment — Submitted filename: Final Review by Pr Juliette TRANCHOT-DIALLO.pdf [file pone.0281612.s002.pdf]

Review by Pr Juliette TRANCHOT-DIALLO

Manuscript Number: PONE-D-22-02970R1
Article Type: Research Article
Full Title: Epidemiology of dengue, chikungunya and Zika virus infection in travellers: a 16-year retrospective descriptive study at a tertiary care centre in Prague, Czech Republic
Short Title: Epidemiology of dengue, chikungunya and Zika in Czech travellers Corresponding **Author**: Vyacheslav Grebenyuk Charles University Second Faculty of Medicine: Univerzita Karlova 2 lekarska fakulta Prague, CZECH REPUBLIC
Keywords: Arboviral infection; Arthropod-borne disease; Travel-associated infection; Traveller

I. Personalized summary of the research work carried out

This is a 16-year prospective study from 2004 to 2019, which took place in a tertiary care center in Prague, Czech Republic. As part of this work, during the relevant study period, the authors propose:

In a first step, to notify the annual number of cases of imported and diagnosed infections, as well as their monthly distribution over the twelve months of the year. These infections concern the three arboviruses dengue, chikungunya and Zika;

In a second step, to define some epidemiological characteristics and clinical features (reasons for travel, region or localities visited, associated clinical manifestations) of patients infected with these three arboviruses already mentioned.

Finally, in the case of dengue fever, to determine the associated serotypes from 2015 to 2017.

II. Summary of the interest of this research article

This study relating to the epidemiological description of cases of infections by dengue, chikungunya and Zika viruses imported to Prague in the Czech Republic through travelers, finds its interest and its relevance, in the context of globalization and the search for solutions at the international level to overcome emerging or re-emerging infectious and vector-borne diseases. These diseases, whether associated or not, are responsible for a heavy burden in terms of morbidity in the world in general and in tropical countries with limited resources in particular.

This work has the merit of giving some visibility on the distribution of infections due to arboviruses and imported from endemic tropical areas to Europe more generally and to the Czech Republic more particularly due to population movements via travel. Indeed, these imported infections such as Dengue, Chikungunya and Zika, which constitute tropical diseases with epidemic potential, require monitoring and scientific research not only at the national level in endemic countries but also internationally in view of their eradication.

In this work, the authors document the regions and countries most visited by patients with DENV, CHIKV, and ZIKV infections, which gives an overview of the impact of these contaminations in terms of public health on a scale of the Czech Republic but more generally suggests the significant impact on a global scale due to travel resulting in significant population movements.
In addition, the results of this work show the need for effective surveillance of populations returning from travel to prevent the spread of these infections in Europe due to the existence of a potentially competent transmission vector.

In countries with limited resources, the clinical manifestations associated with dengue, for example, have similarities to those due to malaria. Also, due to limited resources, in these countries patients are most often treated on the basis of clinical signs alone. Also, the differential diagnosis of infections due to these different arboviruses is not carried out and many of these tropical diseases such as dengue fever are classified in the group of neglected tropical diseases. This present work has the merit of carrying out laboratory research and diagnosis. This makes it possible to give the true incidence of these different vector-borne infectious diseases and to trace their possible international spread through travellers.

III. Problems and comments raised

Some comments have already been taken into account by the previous reviewer, however the following points should be noted.

1#. The case of infection or re-infection or vaccination of the patients in the study are not documented, certainly because of the retrospective nature of the study, which limits the scientific exploitation of the results of this work.

2#. The same is true of the Caucasian or non-Caucasian nature of the study population. Indeed, this information could make it possible to verify whether or not there is a link between the severity of the clinical manifestations and the natural history of the disease in the two study groups.

3#. In the context of this study, patients with, among other things, other imported infections were excluded from the study. However, taking this information into account would have added to the knowledge of co-infections such as malaria-dengue fever and zika-dengue fever, sometimes noted in the literature. This could have documented the correlation between the clinical signs and the share of each of these tropical infections according to the geographical origin of the travel of the infected patients. Something that is often difficult to do in endemic areas due to the lack of laboratory diagnostic means.

4#. The results of figure 1 do not appear explicitly in the results, not even those mentioned in particular in the discussion (line 226-228 in other).

5#. The title of figure 4: "Seasonality of imported arboviral" lacks precision. The seasons are known as for example summer, spring, autumn and winter. In fact, the title could be "January to December distribution of imported arboviral".

IV. Conclusion

Despite the limitations due to insufficiently documented information, certainly linked to the retrospective nature of the study, this work, which meets the publication criteria required by PLOS One, also presents a notorious interest already indicated, for which it deserves to be accepted for publication.
