# Supplementary material for: Epidemiology of dengue, chikungunya and Zika virus infections in travellers: A 16-year retrospective descriptive study at a tertiary care centre in Prague, Czech Republic
Source: PLoS One. 2023 Feb 21;18(2):e0281612. doi: 10.1371/journal.pone.0281612 (PMC9942961; doi:10.1371/journal.pone.0281612)
Supplement: Attachment — Submitted filename: Response to Reviewers.docx [file pone.0281612.s001.docx]

Dear Dr. Roques,

Thank you very much for Your and Reviewer’s opinions on our manuscript and for the suggested revisions. Herewith I am enclosing the rebuttal letter that responds to each point raised.

Kind Regards,

Vyacheslav Grebenyuk

Corresponding author

**Response to Reviewers:**

**Editor**

As requested by the reviewer, try to provide a better clinical description of the various cases you count and in the same time please shorten as far as possible the description by providing synthetic tables as suggested by the reviewer.

Accepted. We have provided detailed clinical description of the cases and shortened the manuscript as far as possible without leaving out the essential parts.

**Editor**

Please indicated in the title that you refer to a Czech Republic institution.

Accepted, the title has been changed.

**Journal Requirements**

Please ensure that your manuscript meets PLOS ONE's style requirements, including those for file naming.

Please ensure that you have an ORCID iD and that it is validated in Editorial Manager.

ORCID iD added and validated in Editorial Manager.

In your Data Availability statement, you have not specified where the minimal data set underlying the results described in your manuscript can be found. PLOS defines a study's minimal data set as the underlying data used to reach the conclusions drawn in the manuscript and any additional data required to replicate the reported study findings in their entirety. All PLOS journals require that the minimal data set be made fully available.

The study’s minimal underlying data set has been made available within the public repository Open Science Framework (osf.io/grw7z DOI 10.17605/OSF.IO/GRW7Z).

**Reviewer #1**

#1 It would be interesting to describe more precisely the clinical manifestations of patients and to use the international classifications for dengue, chikungunya and Zika virus infection.

Accepted. A detailed description of the clinical manifestations is provided, including the international classification for dengue cases (i.e., classic dengue, dengue with warning signs, severe dengue) as per the 2009 WHO classification. Based on symptom duration, chikungunya cases were classified into acute, subacute, and chronic diseases. In the revised manuscript, we have specified that all chikungunya and Zika cases were symptomatic and clinically uncomplicated. We are not aware of any other stable international classification of chikungunya or Zika. The eleventh edition of the International Classification of Diseases contains one designation for Chikungunya virus disease and one for Zika virus disease.

#2 The breakdown between direct and serological diagnosis should be indicated for each arbovirus.

Accepted. We have specified the diagnostic modalities in the text.

#3 For dengue, serological data should make it possible to differentiate primary dengue cases from others.

Accepted, serological data were added to the text. Unfortunately, it is not always possible to differentiate primary and secondary dengue cases as a substantial proportion of Czech travellers is vaccinated against other arboviral diseases (including tick-borne encephalitis and yellow fever), which may lead to cross-reactive IgG positivity. Therefore, we have provided the data on the vaccination status in the revised manuscript. However, due to the study’s retrospective design, precise data on vaccination status or previous arbovirus exposure were not available. We have clarified this point in the discussion.

#4 It would also be interesting to know the dengue serotypes and compare them with the serotypes circulating in the countries visited at the time of travel.

We accept this point and agree that it would add much value to the manuscript. Unfortunately, we do not routinely perform dengue serotyping at our centre due to the limited resources. Nevertheless, there was an effort to establish serotype differentiation in selected dengue patients (i.e., those with PCR positivity and sufficient viral load) from 2015 to 2017. We have added the available data to the manuscript. A comparison with the serotypes circulating at the time is provided in the discussion.

#5 In the end, Guadeloupe is not located in South America but in the Caribbean.

Accepted.
