# Supplementary material for: Epidemiology of dengue, chikungunya and Zika virus infections in travellers: A 16-year retrospective descriptive study at a tertiary care centre in Prague, Czech Republic
Source: PLoS One. 2023 Feb 21;18(2):e0281612. doi: 10.1371/journal.pone.0281612 (PMC9942961; doi:10.1371/journal.pone.0281612)
Supplement: Attachment — Submitted filename: Manuscript (Clean copy).docx [file pone.0281612.s003.docx]

Epidemiology of dengue, chikungunya and Zika virus infections in travellers: a 16-year retrospective descriptive study at a tertiary care centre in Prague, Czech Republic

Milan Trojánek^1,2,3^, Vyacheslav Grebenyuk^1,2^, Zdenka Manďáková^4^, Naděžda Sojková^5^, Hana Zelená^6^, Hana Roháčová^2^, František Stejskal^1,2,7,8^

^1^ Department of Infectious Diseases, 2^nd^ Faculty of Medicine, Charles University, Prague, Czech Republic

^2^ Department of Infectious, Parasitic and Tropical Diseases, University Hospital Bulovka, Prague, Czech Republic

^3^ Department of Infectious Diseases, Institute for Postgraduate Medical Education, Prague, Czech Republic

^4^ Department of Epidemiology of Infectious Diseases, National Institute of Public Health, Prague, Czech Republic

^5^ Department of Clinical Microbiology, University Hospital Bulovka, Prague, Czech Republic

^6^ National Reference Laboratory for Arboviruses, Institute of Public Health in Ostrava, Czech Republic

^7^ Institute of Immunology and Microbiology, 1^st^ Faculty of Medicine, Charles University, Prague, Czech Republic

^8^ Department of Infectious Diseases, Regional Hospital Liberec, Czech Republic

*Corresponding author

Email: [grebenyuk@seznam.cz](mailto:grebenyuk@seznam.cz) (VG)

**Abstract:**

**Introduction.** This study aims to describe the epidemiological characteristics of imported cases of dengue (DEN), chikungunya (CHIK), and Zika virus (ZIKV) infections in Czech travellers.

**Materials and methods.** The single-centre descriptive study retrospectively analysed data of patients with laboratory confirmed DEN, CHIK, and ZIKV infections diagnosed at the Department of Infectious, Parasitic and Tropical Diseases of the University Hospital Bulovka in Prague, Czech Republic from 2004 to 2019.

**Results.** The study included a total of 313 patients with DEN, 30 with CHIK, and 19 with ZIKV infections. Most patients travelled as tourists: 263 (84.0%), 28 (93.3%), and 17 (89.5%), respectively (p=0.337). The median duration of stay was 20 (IQR 14-27), 21 (IQR 14-29), and 15 days (IQR 14-43), respectively (p=0.935). The peak of DEN and ZIKV infections were diagnosed in 2016, while it was it in 2019 for CHIK infection. Most cases of DEN and CHIKV infections were acquired in Southeast Asia:212 (67.7%) and 15 (50%), respectively. While ZIKV infection was most commonly imported from the Caribbean (11; 57,9%).

**Conclusions.** Arbovirus infections represent an increasingly important cause of illness in Czech travellers. Comprehensive knowledge of the specific epidemiological profile of these diseases is an essential prerequisite for good travel medicine practice.

**Introduction**

Over the past five decades, arboviraus infections have emerged from sporadically occurring diseases to a global public health issue due to their expanding geographic distribution, increasing social and economic impact on the population in the endemic regions. Dengue fever is considered the most common arboviraus infection globally, causing over 390 million cases per year [1]. Chikungunya virus (CHIKV) was first described in 1952 and, for the next five decades, caused several minor outbreaks in Africa and Asia. It has reached global significance at the beginning of the 21st century, starting with the 2004 outbreak in Kenya and spreading to islands in the Indian Ocean, India, Southeast Asia, and eventually Latin America in 2013 [2]. Similarly, ZIKV, was first discovered in a Ugandan forest in 1947 and re-emerged on Yap Island in Micronesia in 2007, causing an extensive outbreak of dengue-like illness. Subsequent major outbreaks occurred in French Polynesia in 2013 and the Americas in 2015. It is estimated that ZIKV caused 7.6 million infections in 2016 [3]. Based on the accumulating evidence pointing to an association of ZIKV infection with congenital defects and Guillain-Barré syndrome, on February 1, 2016, the World Health Organisation has declared the rapid spread of the virus as a Public Health Emergency of International Concern [4].

In addition to causing substantial morbidity and socioeconomic disruption in the endemic regions, arbovirus infections pose a significant health risk to travellers visiting these regions for leisure, work, or other purposes. Furthermore, international travel and commerce may contribute to the further spread of these infections outside tropical regions. Sporadic autochthonous infections in parts of Europe and North America have been reported, where a competent mosquito vector is present [5-7]. As travellers from middle- to high-income countries generally have good access to resource-rich specialized medical care, they may serve as sentinels for tropical infections providing extensive data to improve our understanding of their rapidly changing epidemiology.

The main objective of this study was to describe epidemiological characteristics of imported dengue, chikungunya, and Zika virus infections diagnosed at a tertiary centre for travel medicine in the context of global epidemiologic trends. We believe that accumulating and sharing such data is valuable for effective pre-travel consultation practices and good risk management. Moreover, improving physicians’ familiarity with tropical infections may contribute to more efficient diagnostics and improve the quality of care for returned travellers.

**Materials and methods**

This single-centre retrospective descriptive study included all cases of DEN, CHIK, and ZIKV infections in travellers diagnosed at the Department of Infectious, Parasitic and Tropical Diseases of the University Hospital Bulovka in Prague in the years 2004-2019. The department serves as a specialized tertiary care centre for tropical infections in Prague and the Central Bohemian Region, with a catchment area of 2.5 million population. The study was approved by the Ethical Committee of the University Hospital Bulovka (reference number 9214/EK-Z). Informed consent was obtained in all study participants.

**Study subjects**

The study included all patients with laboratory confirmed DEN, CHIK, or ZIKV cases, who presented with compatible symptoms and a history of stay in an endemic region. Patients with other imported or autochthonous infections were excluded from the study.

**Laboratory diagnosis and virology**

DENV infection was diagnosed by the detection of NS1 antigen or viral RNA in acute serum samples, virus-specific IgM and IgG antibodies in acute and convalescent serum samples. CHIKV infection was diagnosed by the detection of viral RNA in serum, virus-specific IgM and subsequently IgG antibodies in acute and convalescent serum samples. A case of ZIKV infection was diagnosed by the detection of viral RNA in blood, urine or sperm or virus-specific IgM and subsequently IgG antibodies and positivity of confirmatory VNT. Virological tests were performed at the Department of Clinical Microbiology of University Hospital Bulovka Prague. Virus-specific IgM and IgG antibodies were detected using commercially available ELISA kits: Dengue Fever Virus IgM Capture ELISA and Dengue indirect IgG ELISA (PanBio, Brisbane, Australia); NovaLisa Chikungunya Virus μ-capture ELISA and Chikungunya Virus IgG capture ELISA (NovaTec Immunodiagnostica, Germany), Anti-Zika IgM and IgG ELISA (Euroimmun, Germany). ZIKV VNT tests were performed at the National Reference Laboratory for Arboviruses (Institute of Public Health in Ostrava). The detection of dengue NS1 antigen was performed using Platelia Dengue NS1 Antigen EIA test (BioRad Laboratories). Viral-specific RNA was detected using commercially available kits: Dengue Virus General-type real-time RT-PCR (Shanghai ZJ, Bio-Tech Co.), Chikungunya Virus Real Time RT-PCR (Shanghai ZJ Bio-Tech, China), Zika virus Real-Time RT-PCR kit (Biolife).

**Clinical data**

The clinical data were retrospectively extracted from the hospital’s electronic medical records. Recorded data included patient demographics (age and sex), travel characteristics (destination, duration, reason for travel, return date), symptoms and timing of illness, clinical manifestations, laboratory findings, treatment, and outcomes. Dengue cases were classified according to the 2009 WHO Classification [8]. Chikungunya cases were classified into acute (symptom duration of ≤14 days), subacute (≤2 months) or chronic disease.

**Statistical methods**

Continuous variables are described by medians with interquartile ranges (IQR). Mann-Whitney or ANOVA tests were used for the comparison of continuous variables between two groups. Categorical variables are represented by absolute frequencies and proportions and analyzed using Fisher’s exact test. A p-value of 0.05 was considered statistically significant. GraphPad PRISM 9.2.0 for Mac was used for all data analysis (GraphPad Software, San Diego California USA, www.graphpad.com).

**Results**

The study included 313 patients with DEN, 30 with CHIK, and 19 with ZIKV infections. The median age was 34 years (IQR 28-41) in DEN patients, 34 (IQR 28-41) in CHIK patients, and 39 years (IQR 31-43) in ZIKV patients (p=0.473). The male to female ratio was 1.19:1, 1.14:1, and 1.11:1 in the DEN, CHIK, and ZIKV subgroups, respectively (p=0.986).

The numbers of cases diagnosed per year are presented in Fig 1. The seasonality of imported DEN, CHIK and ZIKV infections in travellers is presented in Fig 2.

**Figure 1 Numbers of DENV, CHIKV, and ZIKV infections diagnosed per year.**

**Figure 2 Seasonality of imported arboviral infections.**

The reasons for travel are presented in Table 1. No statistically significant difference in the reasons for travel between DEN, CHIK, ZIKV subgroups was identified in this study. The median duration of stay was 20 days (IQR 14-27) in patients with DEN infection, 21 (IQR 14-29), and 15 days (IQR 14-43) in CHIK and ZIKV infection, respectively (p=0.935).

**Table 1. Reason for travel in patients with acute DENV, CHIKV, and ZIKV infections.**

|  | **DENV infection** | | **CHIKV infection** | | **ZIKV infection** | |
| --- | --- | --- | --- | --- | --- | --- |
|  | Number | Percentage | Number | Percentage | Number | Percentage |
| **Reason for travel** |  |  |  |  |  |  |
| **Tourism** | 263 | 84.0 | 28 | 93.3 | 17 | 89.5 |
| **Business** | 34 | 10.9 | 1 | 3.3 | 2 | 10.5 |
| **Visiting friends and relatives (VFR)** | 14 | 4.5 | 1 | 3.3 | 0 | 0.0 |
| **Other** | 2^1^ | 0.6 | 0 | 0.0 | 0 | 0.0 |

^1^ Humanitarian aid (1), study reasons (1)

Southeast Asia was the most common region of stay of the DEN patients (Table 2). While most patients travelled as tourists, the most common destinations among the VFR and business travellers were Vietnam (10/14, 71.4%) and Maledives (23/34, 67.6%), respectively. Twenty-four cases of DEN infection were imported from the Maldives in 2012 and 2013, with the majority (22/24; 91.7%) acquired by construction site workers at a holiday resort on Velaa island in Noonu atoll. Two cases of DEN infection were imported from Sub-Saharan Africa: one from Equatorial Guinea (2011) and one from Tanzania (2014).

**Table 2. Regions and countries most frequently visited by patients with DENV, CHIKV, and ZIKV infections.**

|  | **DENV infection** | | **CHIKV infection** | | **ZIKV infection** | |
| --- | --- | --- | --- | --- | --- | --- |
|  | Number | Percentage | Number | Percentage | Number | Percentage |
| **Southeast Asia** | **212** | **67.7** | **15** | **50.0** | **1** | **5.2** |
| Thailand | 90 | 28.8 | 12 | 40.0 | 1 | 5.2 |
| Indonesia | 71 | 22.7 | 1 | 3.3 | 0 | 0.0 |
| The Philippines | 18 | 5.8 | 0 | 0.0 | 0 | 0.0 |
| Vietnam | 18 | 5.8 | 0 | 0.0 | 0 | 0.0 |
| Cambodia | 8 | 2.6 | 0 | 0.0 | 0 | 0.0 |
| Malaysia | 4 | 1.3 | 1 | 3.3 | 0 | 0.0 |
| **South Asia** | **29** | **9.3** | **3** | **10.0** | **0** | **0.0** |
| India | 18 | 5.8 | 3 | 10.0 | 0 | 0.0 |
| Sri Lanka | 11 | 3.5 | 0 | 0.0 | 0 | 0.0 |
| **Indian Ocean** | **42** | **13.4** | **5** | **16.7** | **1** | **5.2** |
| The Maldives | 41 | 13.1 | 1 | 3.3 | 1 | 5.2 |
| Mauritius | 0 | 0.0 | 4 | 13.3 | 0 | 0.0 |
| **Central America** | **8** | **2.6** | **1** | **3.3** | **5** | **26.3** |
| Costa Rica | 5 | 1.6 | 0 | 0.0 | 1 | 5.2 |
| Mexico | 3 | 1.0 | 1 | 3.3 | 2 | 10.5 |
| Nicaragua | 0 | 0.0 | 0 | 0.0 | 2 | 10.5 |
| **The Caribbean** | **11** | **3.5** | **3** | **10.0** | **12** | **63.2** |
| Cuba | 7 | 2.2 | 0 | 0.0 | 3 | 15.8 |
| Martinique | 0 | 0.0 | 0 | 0.0 | 7 | 36.8 |
| Guadeloupe | 0 | 0.0 | 2 | 6.6 | 1 | 5.2 |
| The Dominican Republic | 1 | 0.3 | 0 | 0.0 | 1 | 5.2 |
| **South America** | **9** | **2.9** | **3** | **10.0** | **0** | **0.0** |
| Brazil | 6 | 1.9 | 2 | 6.6 | 0 | 0.0 |
| Venezuela | 3 | 1.0 | 0 | 0.0 | 0 | 0.0 |
| **Sub-Saharan Africa** | **2** | **0.6** | **0** | 0.0 | **0** | **0.0** |
| Equatorial Guinea | 1 | 0.3 | 0 | 0.0 | 0 | 0.0 |
| Tanzania | 1 | 0.3 | 0 | 0.0 | 0 | 0.0 |

DENV-specific IgM and IgG antibodies were positive in 208/299 (69.6%) and 150/299 (50.2%) patients, respectively. Peripheral blood samples were positive for NS1 antigen or DENV RNA in 263/283 (92.9 %) and 130/166 patients (78.3 %), respectively. The diagnosis of acute dengue in IgG-positive cases was possible based on NS1 antigen detection in combination with PCR positivity in 57/150 cases (38.0%), NS1 positivity and IgM seroconversion in 58/150 (38.7%), NS1 alone in 6/150 (4.0 %), and IgM seroconversion alone in 29/150 (19.3%). No information on previous exposure to arboviruses was available. However, 38/150 (25.3%) of the IgG-positive patients were vaccinated against tick-borne encephalitis (TBE) or yellow fever (YF). Dengue serotypes were available only in selected patients with a high viral load from 2015 to 2017 ( Table 3).

**Table 3: Dengue serotypes detected from 2015 to 2017**

| **Serotypes** | **Years** | **Countries** | **Number of cases** | **Total** |
| --- | --- | --- | --- | --- |
| DENV-1 | 2016 | Indonesia | 4 | 12 |
|  |  | Maldives | 4 |  |
|  |  | Vietnam | 3 |  |
|  |  | Brazil | 1 |  |
| DENV-2 | 2016 | Indonesia | 6 | 13 |
|  |  | Thailand | 3 |  |
|  |  | Cuba | 1 |  |
|  | 2017 | Sri Lanka | 2 |  |
|  |  | Thailand | 1 |  |
| DENV-3 | 2016 | Indonesia | 3 | 5 |
|  |  | Thailand | 1 |  |
|  | 2017 | Indonesia | 1 |  |
| DENV-4 | 2015 | Sri Lanka | 1 | 2 |
|  | 2016 | Indonesia | 1 |  |

The first cases of CHIK infection were diagnosed at department in February and April 2006 in 4 patients who return from Mauritius. In 2009 there were two cases imported from South Asia and two from Southeast Asia. A total of 7 cases were imported from the Americas in 2014 (Columbia, Guadeloupe), 2015 (Jamaica), 2016 (Mexico), and 2018 (Brazil). However, most CHIKV cases (12/30; 40.0%) were imported from Thailand in 2019. Acute sera (<14 days from symptom onset) were available in 16/30 (53.3%) patients. CHIKV-specific IgM and IgG antibodies were detected in 9/16 (56.2%) and 4/16 (25.0%) of acute cases, respectively. Of the 3 patients tested peripheral blood CHIKV RNA was positive in 2. In all cases with negative acute serology subsequent IgG seroconversion was detected in convalescent sera. In subacute cases IgM were positive in 12/14 (85.7%) and IgG in 13/14 (92.9%).

Zika virus infection was first diagnosed at our study site in January 2016 in two patients who returned from Martinique. Fourteen cases were diagnosed in 2016, and all were imported from the Caribbean or Central America. In the following year, 3 cases of ZIKV infection were diagnosed in patients returning from Cuba, and one case in a patient returning from the Maldives. The last case of ZIKV infection was diagnosed in November 2019 in a patient who stayed in Thailand. ZIKV-specific IgM and IgG antibodies were detected in 12/19 (63.1%) and 8/19 (42.1%) patients, respectively. VNT confirmation was performed in 16/19 (84.2%) cases with median titre of 1:32 (IQR 1:64 to 1:16). ZIKV RNA was detected by RT-PCR in various samples from 7 patients: blood in 2/13 (15.4%), urine in 6/15 (40.0%), and semen in 3/5 (60.0%). In two cases ZIKV-RNA was detected in sperm samples collected more than 60 days from symptom onset.

The median period from symptoms onset to presentation was 4 days (IQR 3-6) in patients with DENV, 9 days (IQR 5-23), and 7 days (IQR 3-19) in CHIK and ZIKV infections, respectively (p<0.001). The most common clinical manifestations of DEN, CHIK and ZIKV infections are presented in Table 3. The median duration of fever was five days (IQR 4-6) in dengue, four days (IQR 3-5) in chikungunya, and five days (IQR 4-6) in Zika virus infection (p=0.035). Joint pain was reported by all patients with CHIK infection. Most affected were small joints of hands or feet (26/30; 86.7%), ankles (12/30; 40.0%), and knees (12/30; 40.0%). Nineteen patients suffered chronic arthritis lasting over 2 months (63.3%). All Zika virus disease cases were symptomatic, and most reported either low-grade fever (8/19, 42.1%) or no fever (3/19 15.8%).

**Table 4. Clinical manifestations of dengue, chikungunya and Zika.**

| **Signs and symptoms** | **DEN** | | **CHIK** | | **ZIKV** | | **P value** |
| --- | --- | --- | --- | --- | --- | --- | --- |
|  | N | % | N | % | N | % |  |
| **Fever >38°C** | 311/313 | 99.4 | 30/30 | 100.0 | 8/19 | 42.1 | **<0.001** |
| **Rash** | 225/313 | 71.9 | 26/30 | 86.7 | 18/19 | 94.7 | **0.023** |
| **Headache** | 223/313 | 71.2 | 15/30 | 50.0 | 14/19 | 73.7 | **0.050** |
| **Joint pain** | 189/313 | 60.4 | 30/30 | 100.0 | 14/19 | 73.7 | **<0.001** |
| **Joint swelling** | 0/313 | 0.0 | 19/30 | 63.3 | 8/19 | 42.1 | **<0.001** |
| **Muscle pain** | 214/313 | 68,4 | 14/30 | 46.7 | 6/19 | 31.6 | **<0.001** |
| **Abdominal pain** | 36/313 | 11.5 | 1/30 | 3.3 | 1/19 | 5.3 | 0.137 |
| **Diarrhoea** | 91/313 | 29.1 | 7/30 | 23.3 | 8/19 | 42.1 | 0.363 |
| **Vomiting** | 49/313 | 15.7 | 2/30 | 6.7 | 0/19 | 0.0 | **0.027** |
| **Pruritus** | 137/313 | 43.8 | 7/30 | 23.3 | 6/19 | 31.6 | 0.064 |
| **Dysgeusia** | 57/313 | 18.2 | 2/30 | 6.7 | 0/19 | 0.0 | **0.011** |
| **Mucosal bleeding** | 49/313 | 15.7 | 0/30 | 0.0 | 0/19 | 0.0 | **0.001** |
| **Dehydration** | 25/313 | 8.0 | 2/30 | 6.7 | 0/19 | 0.0 | 0.557 |
| **Conjunctivitis** | 34/313 | 10.9 | 4/30 | 13.3 | 8/19 | 42.1 | **0.011** |
| **Pharyngitis** | 83/313 | 26.5 | 5/30 | 16.7 | 4/19 | 21.1 | 0.449 |
| **Hepatomegaly** | 26/313 | 8.3 | 2/30 | 6.7 | 1/19 | 5.3 | 0.781 |
| **Splenomegaly** | 12/313 | 3.8 | 0/30 | 0.0 | 0/19 | 0.0 | 0.382 |
| **Lymphadenopathy** | 43/313 | 13.7 | 5/30 | 16.7 | 6/19 | 31.6 | 0.102 |

A total of 108/313 (34.5%) patients with DEN and 6/30 (20.0%) with CHIK infections were treated as inpatients (p=0.154). All patients with ZIKV infection were treated as outpatients. Dengue warning signs were present in 73/313 (23.3%) of cases. One patient (0.3%) required intensive care for severe dengue with plasma leakage, hypotension, pleural effusion, and massive ascites. All acute chikungunya and Zika virus infection cases presented with uncomplicated disease with no alarm signs. There were no lethal outcomes.

**Discussion**

The presented study describes the epidemiological and clinical characteristics of patients with imported dengue, chikungunya, and Zika virus infections diagnosed in a tertiary care centre in Prague, Czech Republic. With 362 patients, this constitutes numerically one of the most extensive studies of imported arboviral infections in Central Europe, providing valuable data for appreciation of the current epidemiological trends in the region. Substantial increments in the incidence of imported DENV, CHIKV, and ZIKV were observed from 2016 to 2019, reflecting the dramatic increase in the global burden of these diseases in recent years. An important contribution of this report is the direct comparison of epidemiological characteristics of the three most common imported arboviral infections, which allows for pinpointing clinically relevant distinctions between the three otherwise very similar diseases.

DENV infection was consistently diagnosed in returned travellers during the whole study period, while significant outbreaks in popular tourist destinations were reflected by sharp increases in imported cases demonstrated in 2013, 2016, and 2019. In contrast, a significant risk of chikungunya or Zika acquisition was present only during major epidemics and virtually absent in all other times. Travellers from high-income countries may thus serve as sentinels for tropical infections. At the same time, there was also an apparent shift of the overall case burden to Southeast and South Asia during the most recent years. These changes are particularly important to European travellers, as they represent the largest and still growing proportion of international tourist arrivals to these regions [9]. Furthermore, as demonstrated previously, the possibility of sexual transmission of arboviral infections poses an additional risk of spreading the disease to non-travellers [10].

DEN infection remains the most important imported arboviral disease in Czech travellers, as demonstrated in a ten-year retrospective study previously conducted in our centre [11]. The incidence peaks were observed in 2016 and 2019, reflecting higher transmission rates of the virus in the Americas and especially Southeast Asia [12,13]. The number of cases peaked in mid-summer, late autumn, and early spring, similar to the patterns observed in other European travellers [14]. Apart from tourists, who represented most DENV cases, there was a significant proportion of business travellers. Of particular interest are the 22 dengue cases diagnosed in Czech workers at the construction site in the Maldives [15]. Outbreaks like this indicate a potential target for optimisation of the pre-travel counselling practices. Similar to the findings in other European travellers, dengue cases imported by VFR and travellers from Sub-Saharan Africa represented very small proportions in this study [14,16,17]. Dengue, however, should still be considered a diagnostic possibility in febrile patients returning from Africa, especially when malaria is excluded. Furthermore, travellers who visit friends and relatives in dengue-endemic regions are at higher risk of severe dengue due to possible prior exposure to a different DENV serotype [18]. All four serotypes are co-circulating in hyperendemic regions with cyclic re-emergence and dominance of specific serotypes [18-21]. The most common serotypes found in this study were DENV-1 and DENV-2. These findings are probably more reflective of the specific travelling patterns in our patients than the current epidemiological situation in the visited countries. Most Czech patients travel to Southeast Asia as tourists and typically visit coastal cities and surrounding islands (e.g., Krabi, Bali, Ko Samui etc.) [10]. Due to the significant Vietnamese minority in the Czech Republic, we see many VFR among travellers returning from Vietnam. These are more likely to visit distant areas and rural regions. Interestingly, the only serotype isolated from travellers returning from Vietnam was DENV-1, which was the predominant serotype during the 2017 outbreak [22]. DENV-1 was also found in patients returning from the Maldives. The country experienced two large outbreaks of dengue in the recent decade. Only DENV-1 was detected in 2011, however, DENV-2 was reported in a Japanese couple during the 2018 outbreak [23-24]. This finding suggests a recent shift in the dominant serotype with an increased risk of severe dengue, particularly in non-immune populations (e.g., children, travellers) [25]. Due to the study’s retrospective design, it was impossible to differentiate all primary and secondary dengue cases reliably. Moreover, previous exposure to other arboviruses or vaccination may lead to cross-reactive IgG positivity. The proportion of IgG-positive patients who reported previous vaccination against TBE or YF was 25%.

Clinically dengue cases presented with acute febrile illness often accompanied by rash, headache, myalgias and arthralgias. However, skin involvement was less common than in chikungunya and Zika patients and absent in 28%. Dengue warning signs were present in 23% and there was one case of severe dengue.

The first chikungunya cases were imported from Mauritius in 2006 following the large outbreak on the islands of the Indian Ocean [27]. The incidence of CHIKV peaked in 2019 following another outbreak in Thailand [28]. Southeast Asia has become the most common region of infection in European travellers due to its popularity among tourists and the high burden of chikungunya cases in the area [29]. CHIK cases tend to present late due to the relatively short duration of acute febrile phase in CHIKV infection and the high incidence of persistent subacute complications [30,31]. Acute chikungunya was characterized by fever, rash and arthralgias. Joint swelling and chronic arthritis developed in 63%.

The cases of imported ZIKV infection in Europe peaked in 2016 following the large outbreak in the Americas. Since then, they have been declining at similarly rapid rates, as are demonstrated in our study [5]. Notably, all Zika cases were symptomatic, typically presenting with low-grade fever and rash. Arthralgias were reported by 74%, however, in contrast to chikungunya, joint swelling was significantly less common, and there was no progression to chronic arthritis. Other common features included conjunctivitis, pharyngitis, lymphadenopathy, and diarrhoea.

Despite the overall decrease in ZIKV imported to Europe, the number of infections acquired in Southeast Asia has increased severalfold since 2016 [5]. Most of these cases were infected in Thailand, the most popular tropical destination among Czech tourists. The rise in imported Zika cases may be partially accounted for by improved awareness of the disease and better availability of diagnostic methods since the association with congenital defects and Guillain-Barré syndrome was reported. Although the virus has been present in Southeast Asia for over 50 years, the true incidence of human infections is unknown. ZIKV is assumed to have primarily enzoonotic circulation in the region with only occasional spillover to humans [32].  No major outbreak has occurred to date, and only a few scattered cases have been reported in the literature [33]. The potential re-emergence of ZIKV in Southeast Asia has significant implications for travel medicine in Europe. Women of childbearing age and their partners travelling to the region should be counselled on the appropriate preventive measures, including mosquito bite precautions. Practitioners should also provide appropriate post-travel advice to returning travellers seeking care due to symptomatic disease, as well as anyone with significant exposure, particularly when planning pregnancy. Notably, we have detected ZIKV RNA in sperm samples of two patients collected more than 60 days from symptom onset. The issue of prolonged viral shedding in semen and vaginal secretions with the potential of sexual transmission for up to several months after infection should be addressed in all patients returning from the areas with an ongoing ZIKV transmission.

**Conclusions**

Tropical arboviral infections are among the most important causes of travel-related morbidity. In addition to being the most popular tourist destination among Europeans, Southeast Asia has recently become the region with the highest risk of acquiring tropical arboviral infections. DENV infection represents a constant threat present during epidemics, as well as during the interim periods. Although travellers to Asia and Latin America are disproportionally affected, the risk of dengue is significant throughout the tropical zone, including Sub-Saharan Africa. Although CHIKV and ZIKV infections remain relatively rare causes of illness in Czech travellers, surges in imported cases invariably occur during major outbreaks in popular tourist destinations. Tourists may thus serve as sentinels for epidemiological surveillance of these diseases. With climate change and the steady increase in global travel and migration, there is a growing potential for autochthonous spread of tropical infections in parts of Europe, as well as future arboviral epidemics all over the world. For this reason, physicians should become more familiar with existing and future arboviral threats.

**Acknowledgements**

None.

**References**

[1] Bhatt S, Gething PW, Brady OJ, Messina JP, Farlow AW, Moyes CL, et al. The global distribution and burden of dengue. Nature. 2013 Apr 25;496(7446):504-7. doi: 10.1038/nature12060.

[2] Silva JVJ Jr, Ludwig-Begall LF, Oliveira-Filho EF, Oliveira RAS, Durães-Carvalho R, Lopes TRR, et al. A scoping review of Chikungunya virus infection: epidemiology, clinical characteristics, viral co-circulation complications, and control. Acta Trop. 2018 Dec;188:213-224. doi: 10.1016/j.actatropica.2018.09.003.

[3] GBD 2016 Disease and Injury Incidence and Prevalence Collaborators. Global, regional, and national incidence, prevalence, and years lived with disability for 328 diseases and injuries for 195 countries, 1990-2016: a systematic analysis for the Global Burden of Disease Study 2016. Lancet. 2017 Sep 16;390(10100):1211-1259. doi: 10.1016/S0140-6736(17)32154-2. Erratum in: Lancet. 2017 Oct 28;390(10106):e38.

[4] Basile K, Kok J, Dwyer DE. Zika virus: what, where from and where to? Pathology. 2017;49(7):698-706. doi: 10.1016/j.pathol.2017.08.002.

[5] European Centre for Disease Prevention and Control. Zika virus disease - Annual epidemiological report for 2019. European Centre for Disease Prevention and Control. 2021 Mar 2021 [Cited 2021 Oct 10]. Available from: <https://www.ecdc.europa.eu/sites/default/files/documents/AER-Zika-2019.pdf>

[6] European Centre for Disease Prevention and Control. Autochthonous transmission of dengue virus in mainland EU/EEA, 2010-present. European Centre for Disease Prevention and Control. 2021. Available from: <https://www.ecdc.europa.eu/en/all-topics-z/dengue/surveillance-and-disease-data/autochthonous-transmission-dengue-virus-eueea>

[7] Thomas DL, Santiago GA, Abeyta R, Hinojosa S, Torres-Velasquez B, Adam JK, et al. Reemergence of Dengue in Southern Texas, 2013. Emerg Infect Dis. 2016 Jun;22(6):1002-7. doi: 10.3201/eid2206.152000.

[8] Dengue: Guidelines for Diagnosis, Treatment, Prevention and Control: New Edition. Geneva: World Health Organization; 2009.

[8] World Tourism Organization. International Tourism Highlights, 2019 Edition. Madrid: UNWTO; 2019. DOI: <https://doi.org/10.18111/9789284421152>

[9] Frank C, Cadar D, Schlaphof A, Neddersen N, Günther S, Schmidt-Chanasit J, et al. Sexual transmission of Zika virus in Germany, April 2016. Euro Surveill. 2016 Jun 9;21(23). doi: 10.2807/1560-7917.ES.2016.21.23.30252.

[10] Trojánek M, Maixner J, Sojková N, Kynčl J, Roháčová H, Marešová V, Stejskal F. Dengue fever in Czech travellers: A 10-year retrospective study in a tertiary care centre. Travel Med Infect Dis. 2016 Jan-Feb;14(1):32-38. doi: 10.1016/j.tmaid.2015.06.005.

[11] Cogan JE. Dengue and severe dengue. World Health Organization. 2021. Available from: <https://www.who.int/news-room/fact-sheets/detail/dengue-and-severe-dengue>

[12] Pan American Health Organization. Dengue Cases; 2021[cited 2021 July 26]. Database: Pan American Health Organization [Internet]. Available from: <https://www3.paho.org/data/index.php/en/mnu-topics/indicadores-dengue-en/dengue-nacional-en/252-dengue-pais-ano-en.html>

[13] European Centre for Disease Prevention and Control. Dengue – Annual epidemiological report for 2019. European Centre for Disease Prevention and Control. 2021 Apr 14 [Cited 2021 Oct 18]. Available from: https://www.ecdc.europa.eu/sites/default/files/documents/AER-dengue-2019.pdf

[14] Trojánek M, Tomíčková D, Roháčová H, Kosina P, Gebouský J, Dvořák J, et al. Případy horečky dengue u českých pracovníků zaměstnaných na Maledivách [Dengue fever cases in Czech workers returning from the Maldives]. Epidemiol Mikrobiol Imunol. 2013 Sep 62(3):100-5.

[15] Tavakolipoor P, Schmidt-Chanasit J, Burchard GD, Jordan S. Clinical features and laboratory findings of dengue fever in German travellers: A single-centre, retrospective analysis. Travel Med Infect Dis. 2016 Jan-Feb;14(1):39-44. doi: 10.1016/j.tmaid.2016.01.007.

[16] Teichmann D, Göbels K, Niedrig M, Grobusch MP. Dengue virus infection in travellers returning to Berlin, Germany: clinical, laboratory, and diagnostic aspects. Acta Trop. 2004 Mar;90(1):87-95. doi: 10.1016/j.actatropica.2003.11.004.

[17] Shukla R, Ramasamy V, Shanmugam RK, Ahuja R, Khanna N. Antibody-Dependent Enhancement: A Challenge for Developing a Safe Dengue Vaccine. Front Cell Infect Microbiol. 2020 Oct 22;10:572681. doi: 10.3389/fcimb.2020.572681.

[18] European Medicines Agency. Dengvaxia. European Medicines Agency. 2020. Available from: <https://www.ema.europa.eu/en/medicines/human/EPAR/dengvaxia>

[19] Phadungsombat J, Lin MY, Srimark N, Yamanaka A, Nakayama EE, Moolasart V, Suttha P, Shioda T, Uttayamakul S. Emergence of genotype Cosmopolitan of dengue virus type 2 and genotype III of dengue virus type 3 in Thailand. PLoS One. 2018 Nov 12;13(11):e0207220. doi: 10.1371/journal.pone.0207220.

[20] Utama IMS, Lukman N, Sukmawati DD, Alisjahbana B, Alam A, Murniati D, Utama IMGDL, Puspitasari D, Kosasih H, Laksono I, Karyana M, Karyanti MR, Hapsari MMDEAH, Meutia N, Liang CJ, Wulan WN, Lau CY, Parwati KTM. Dengue viral infection in Indonesia: Epidemiology, diagnostic challenges, and mutations from an observational cohort study. PLoS Negl Trop Dis. 2019 Oct 21;13(10):e0007785. doi: 10.1371/journal.pntd.0007785.

[21] Nonyong P, Ekalaksananan T, Phanthanawiboon S, Aromseree S, Phadungsombat J, Nakayama EE, Shioda T, Sawaswong V, Payungporn S, Thaewnongiew K, Overgaard HJ, Bangs MJ, Alexander N, Pientong C. Dengue virus in humans and mosquitoes and their molecular characteristics in northeastern Thailand 2016-2018. PLoS One. 2021 Sep 14;16(9):e0257460. doi: 10.1371/journal.pone.0257460.

[22] Hamel R, Surasombatpattana P, Wichit S, Dauvé A, Donato C, Pompon J, Vijaykrishna D, Liegeois F, Vargas RM, Luplertlop N, Missé D. Phylogenetic analysis revealed the co-circulation of four dengue virus serotypes in Southern Thailand. PLoS One. 2019 Aug 15;14(8):e0221179. doi: 10.1371/journal.pone.0221179.

[23] Takemura T, Nguyen CT, Pham HC, Nguyen TT, Hoang VMP, Nguyen LKH, Nabeshima T, Nguyen TTT, Le TQM, Moi ML, Morita K, Hasebe F. The 2017 Dengue virus 1 outbreak in northern Vietnam was caused by a locally circulating virus group. Trop Med Health. 2022 Jan 4;50(1):3. doi: 10.1186/s41182-021-00386-0.

[24] Abdulla AA, Rasheeda F, Ahmed IN, Aboobakur M. An evaluation of the surveillance system for dengue virus infections in Maldives. WHO South East Asia J Public Health. 2014 Jan-Mar;3(1):60-68. doi: 10.4103/2224-3151.206886.

[25] Mutoh Y, Moriya A, Yasui Y, Saito N, Takasaki T, Hiramatsu S, Izuchi T, Umemura T, Ichihara T. Two Cases of Dengue Virus Type 2 (DENV-2) Infection in a Japanese Couple Returning from the Maldives during the 2018 Dengue Outbreak. Jpn J Infect Dis. 2020 Jan 23;73(1):58-60. doi: 10.7883/yoken.JJID.2019.153.

[26] Endy TP, Nisalak A, Chunsuttiwat S, Libraty DH, Green S, Rothman AL, Vaughn DW, Ennis FA. Spatial and temporal circulation of dengue virus serotypes: a prospective study of primary school children in Kamphaeng Phet, Thailand. Am J Epidemiol. 2002 Jul 1;156(1):52-9. doi: 10.1093/aje/kwf006.

[27] Kariuki Njenga M, Nderitu L, Ledermann JP, Ndirangu A, Logue CH, Kelly CHL, et al. Tracking epidemic Chikungunya virus into the Indian Ocean from East Africa. J Gen Virol. 2008 Nov;89(Pt 11):2754-2760. doi: 10.1099/vir.0.2008/005413-0.

[28] Khongwichit S, Chansaenroj J, Thongmee T, Benjamanukul S, Wanlapakorn N, Chirathaworn C, Poovorawan Y. Large-scale outbreak of Chikungunya virus infection in Thailand, 2018-2019. PLoS One. 2021 Mar 10;16(3):e0247314. doi: 10.1371/journal.pone.0247314.

[29] European Centre for Disease Prevention and Control. Chikungunya virus disease - Annual epidemiological report for 2019. European Centre for Disease Prevention and Control. 2021 Apr 14 [Cited 2021 Oct 19]. Available from: <https://www.ecdc.europa.eu/sites/default/files/documents/chikungunya-annual-epidemiological-report-2019.pdf>

[30] Taubitz W, Cramer JP, Kapaun A, Pfeffer M, Drosten C, Dobler G, et al. Chikungunya fever in travelers: clinical presentation and course. Clin Infect Dis. 2007 Jul 1;45(1):e1-4. doi: 10.1086/518701.

[31] Rodríguez-Morales AJ, Cardona-Ospina JA, Fernanda Urbano-Garzón S, Sebastian Hurtado-Zapata J. Prevalence of Post-Chikungunya Infection Chronic Inflammatory Arthritis: A Systematic Review and Meta-Analysis. Arthritis Care Res (Hoboken). 2016 Dec;68(12):1849-1858. doi: 10.1002/acr.22900.

[32] Wikan N, Smith DR. Zika virus from a Southeast Asian perspective. Asian Pac J Trop Med. 2017 Jan;10(1):1-5. doi: 10.1016/j.apjtm.2016.11.013.

[33] Wiwanitkit V. The current status of Zika virus in Southeast Asia. Epidemiol Health. 2016 Jun 16;38:e2016026. doi: 10.4178/epih.e2016026.

[34] Nguyen CT, Moi ML, Le TQM, Nguyen TTT, Vu TBH, Nguyen HT, et al. Prevalence of Zika virus neutralizing antibodies in healthy adults in Vietnam during and after the Zika virus epidemic season: a longitudinal population-based survey. BMC Infect Dis. 2020 May 11;20(1):332. doi: 10.1186/s12879-020-05042-2.
