# Supplementary material for: Epidemiology of dengue, chikungunya and Zika virus infections in travellers: A 16-year retrospective descriptive study at a tertiary care centre in Prague, Czech Republic
Source: PLoS One. 2023 Feb 21;18(2):e0281612. doi: 10.1371/journal.pone.0281612 (PMC9942961; doi:10.1371/journal.pone.0281612)
Supplement: Attachment — Submitted filename: Response to Reviewers.docx [file pone.0281612.s004.docx]

**Submission** PONE-D-22-02970R1

**Response to Reviewers**

Dear Dr. Roques,

Thank you for giving us the opportunity to submit a revised draft of the manuscript “Epidemiology of dengue, chikungunya and Zika virus infections in travellers: a 16-year retrospective descriptive study at a tertiary care centre in Prague, Czech Republic” for publication in PLOS One. We appreciate the time and effort that you and the reviewers dedicated to providing feedback on our manuscript and are very grateful for the valuable improvements to our paper. We have incorporated all the suggestions made the reviewers and revised the manuscript for English language and grammar. All changes are highlighted within the manuscript using tracked changes. Please see below, in blue, for a point-by-point response to the reviewers’ comments.

Sincerely,

Vyacheslav Grebenyuk

Corresponding author

**Reviewer Comments:**

**Reviewer #2**

1#. The case of infection or re-infection or vaccination of the patients in the study are not documented, certainly because of the retrospective nature of the study, which limits the scientific exploitation of the results of this work.

Accepted. We have reviewed the medical records of all the dengue cases included in the study and did not find any patient, who reported previous infection with dengue or other tropical arbovirus. Obviously, some cases might have been missed as clinicians do not always explicitly ask the patients about previous infections. However, the vast majority of patients were short-term tourists of Czech nationality. Secondary infections in similar populations are relatively uncommon. In addition, only 111 patients with dengue reported previous stay in an endemic region. Although a tetravalent dengue vaccine (Dengvaxia) has been licensed in many countries, it has not been registered in the Czech Republic up until December 2018 and is still not recommended for use in travellers.

2#. The same is true of the Caucasian or non-Caucasian nature of the study population. Indeed, this information could make it possible to verify whether or not there is a link between the severity of the clinical manifestations and the natural history of the disease in the two study groups.

Accepted, data on the origin of the study participants have been added to the manuscript. No significant differences in the severity of clinical manifestations or natural history of the disease were found. However, the study may be underpowered to show an association as only a minority of patients were non-Caucasians.

3#. In the context of this study, patients with, among other things, other imported infections were excluded from the study. However, taking this information into account would have added to the knowledge of co-infections such as malaria-dengue fever and zika-dengue fever, sometimes noted in the literature. This could have documented the correlation between the clinical signs and the share of each of these tropical infections according to the geographical origin of the travel of the infected patients. Something that is often difficult to do in endemic areas due to the lack of laboratory diagnostic means.

We understand this point and agree with the Reviewer, that it would be interesting and informative to report co-infections with two or more different imported pathogens. However, as we noted in the previous report from our centre (Grebenyuk V, et al. Epidemiol Mikrobiol Imunol. 2021), endemic tropical infections are responsible for a relatively small fraction of the overall morbidity in Czech travellers returning from tropical destinations. Particularly chikungunya and Zika represent rare infections comprising <1% of all travel-associated health problems. Considering this, the likelihood of a co-infection with another endemic pathogen should be very low. Moreover, Zika serology has become available in our centre only since 2016, although the disease has been known to cause outbreaks for almost a decade before that. The rates of imported dengue and malaria are considerably higher making co-infection by these two pathogens more likely. However, a 2018 systematic review (Salam N, et al. BMC Public Health, 2018) found only 2 cases diagnosed in Europe (Spain and France). After carefully reviewing all cases DEN, CHIK and ZIKV infections diagnosed at our centre during the study period we found no co-infections. The Methodology has been revised accordingly.

4#. The results of figure 1 do not appear explicitly in the results, not even those mentioned in particular in the discussion (line 226-228 in other).

Accepted, these results are now mentioned in the text.

5#. The title of figure 4: "Seasonality of imported arboviral" lacks precision. The seasons are known as for example summer, spring, autumn, and winter. In fact, the title could be "January to December distribution of imported arboviral".

Accepted, the title has been changed as suggested.

**Reviewer #3:**

This manuscript describes the epidemiological characteristics of imported human arbovirus infections (dengue, chikungunya and Zika virus) in a tertiary care centre in Prague, Czech Republic in 2004 to 2019. There is strength in the work, with particularly contribution evident in providing an indication of the situation of arbovirus infections in Prague. However, the work does not accurately reflect the epidemiological situation of the infections neither on local (Prague) nor national (Czech Republic) level. The author did not mention whether these records represent all the arbovirus notifications reported in the Prague/ Czech. So, the scope of the work deals with ‘local’ topic, rather than genuinely ‘international’ one. I encourage authors to publish this paper as a national report or in a national journal after improving the manuscript.

The topic is interesting, and there are the foundations for a useful study. However, there is a lack of proper structure (especially in background and discussion) and detail about the knowledge gap, aims and objectives the methodology (collecting and recording the notifications). The analysis of data is also very much underdone (i.e. overly simple analysis has been performed). Further, the manuscript needs extensive revision for language and grammar
to improve the readability.

The author did not discuss the risk of local transmission of these infections in Czech Republic by way of infected travellers and evaluate the other risk factors that might coincide in time or space to trigger a local outbreak. The risk of local transmission is always more critical the than the importation risks of arboviruses. A better contextualise the work in terms of public health risk would greatly improve the value of the work. Hopefully the author can remedy these points and improve the manuscript.

We appreciate the feedback provided by the Reviewer and are grateful for the valuable points raised. The single-centre nature of the study is a significant limitation, and the presented data do not represent all cases imported to the Czech Republic. To compensate for this shortcoming, we have requested the National Institute of Public Health to provide complete data on arbovirus epidemiology. These data have been added to the Discussion.

The Healthcare system in the Czech Republic is highly centralized, and many rare diseases tend to be concentrated in a few large institutions. The Department of Infectious Diseases of the University Hospital Bulovka is the principal referral centre for infectious diseases not only for Prague and the Central Bohemian region but, effectively, for the whole country. Although this work does not represent all the infections notified in the Czech Republic, it does include the vast majority of them (especially in the case of chikungunya and Zika). Thus, we believe our data may reflect the epidemiological situation in the country. Furthermore, since there is a scarcity of data on tropical arboviral infections imported to Central and Eastern Europe, the authors maintain that the study provides valuable insight into the arbovirus epidemiology in the whole region and may prompt more research on the topic in the neighbouring countries.

Based on the Reviewer’s suggestions, we have made some changes to the manuscript. The risk of local transmission and the introduction of the mosquito vectors were added to the Introduction. The paragraph discussing the risk of sexual transmission of ZIKV has been revised in the Discussion. Also, the aim, objectives and knowledge gap have been described in more detail in the Introduction and Discussion. The methodology was likewise revised following the provided comments. Results and Discussion were divided into subsections to improve readability. Finally, the manuscript has been revised for the English Language by a post-doc in English Language and Linguistics.
